# The Role of the Carboxyl-Terminal Sequence of Tau and MAP2 in the Pathogenesis of Dementia

**DOI:** 10.3389/fnmol.2016.00158

**Published:** 2016-12-27

**Authors:** Ce Xie, Tomohiro Miyasaka

**Affiliations:** ^1^College of Basic Medical Sciences, Dalian Medical UniversityDalian, China; ^2^Department of Neuropathology, Faculty of Life and Medical Sciences, Doshisha UniversityKyotanabe, Japan

**Keywords:** tau, tauopathy, microtubule-associated protein 2, MAP2, inclusion, Alzheimer’s disease

## Abstract

Dementia includes several diseases characterized by acquired and irreversible brain dysfunctions that interfere with daily life. According to the etiology, dementia can be induced by poisoning or metabolic disorders, and other cases of dementia have a clear pathogenesis. However, half of neurodegenerative diseases have an unclear pathogenesis and etiology. Alzheimer’s disease (AD), Lewy body dementia and frontal-temporal dementia are the three most common types of dementia. These neurodegenerative diseases are characterized by the appearance of the following specific protein inclusions: amyloid beta and tau in AD; α-synuclein in Lewy body dementia; and tau, TDP-43, or FUS in frontal-temporal dementia. Thus far, studies on the pathogenesis of dementia mainly focus aberrant inclusions formed by the aforementioned proteins. As a historically heavily studied protein tau is likely to be associated with the pathogenesis of several neurodegenerative diseases that cause dementia. The role of tau in neurodegeneration has been unknown for many years. However, both pathological and genetic analyses have helped tau become gradually recognized as an important factor in the pathogenesis of tauopathy. Currently, especially in the field of AD, tau is attracting more attention and is being considered a potential target for drug development. In this review article, previously discovered biochemical and pathological features of tau are highlighted, and current opinions regarding the neurotoxicity of tau are summarized. Additionally, we introduce key amino acid sequences responsible for tau neurotoxicity from our studies using transgenic *Caenorhabditis elegans*. Finally, a new hypothesis regarding the roles of microtubule-associated protein 2 (MAP2) and tau in the pathogenesis of tauopathy is discussed.

## Tau and Tauopathy

Tau is a neuronal microtubule-associated protein and is primarily distributed in axons. It has been suggested that the major function of tau is the stabilization of microtubules, but additional studies on tau-knockout mice may reveal previously unknown functions in the future (Kimura et al., 2013). The gene encoding tau in humans is *MAPT*, which is located on the long arm of chromosome 17 (17q21). In the human adult brain, translated tau mRNA is spliced into six isoforms: 0N3R, 0N4R, 1N3R, 1N4R, 2N3R and 2N4R. In the peripheral nervous system, the longer tau isoform is expressed because of the exon 4a insertion. The microtubule-binding domains (MTBD) are located on the carboxyl terminal of tau and consist of three or four 31 amino acid repeats (R; Mandelkow and Mandelkow, 2012; West and Bhugra, 2015).

The relationship between tau and neurodegenerative diseases was recognized through the characterization of the components of neurofibrillary tangles (NFT). NFT are one of the major pathological hallmarks in brains affected by neurodegenerative diseases. Studies using human brains suggested that the frequency and distribution of NFT are highly correlated with neuronal loss and clinical symptoms (Arriagada et al., 1992; Delacourte et al., 1999). In addition, since 1998, more than 40 mutations in the tau gene were identified in frontotemporal dementia with parkinsonism-17 (FTDP-17), which is a hereditary disease (Mandelkow and Mandelkow, 2012). Therefore, aberrant tau protein may have a causal relationship with the pathogenesis of neurodegeneration.

Tau-inclusions are observed in the brains of patients affected by several neurodegenerative diseases; therefore, these diseases are termed tauopathies. One hereditary tauopathy is FTDP-17, which is caused by mutations in *MAPT* (Goedert et al., 1998; Spillantini and Goedert, 2000). Sporadic tauopathies include several neurodegenerative diseases, such as Alzheimer’s disease (AD), frontotemporal dementia, cortical basal degeneration, progressive supranuclear palsy and others (Spillantini and Goedert, 2013).

AD is the most common neurodegenerative disease that causes senile dementia. The pathogenesis of AD is still largely unknown (Mandelkow and Mandelkow, 2012; West and Bhugra, 2015). There are three major pathological characteristics of AD, including senile plaques formed by amyloid beta, NFT formed by tau and neuronal loss. Beta and gamma secretases cleave amyloid beta precursor protein (APP) to produce amyloid beta. Based on the analysis of human brain tissue, senile plaques are detected earlier than NFT in the cerebral neocortex (Braak and Braak, 1997; Hardy and Selkoe, 2002).

The importance of senile plaques in the pathogenesis of AD was recognized by genetic analysis. Linkage studies of familial AD revealed mutations in *APP*, *PSEN1 (PS1)* and *PSEN2 (PS2*; Hardy and Selkoe, 2002). Meanwhile, *PS1* and *PS2* were identified as key components of gamma-secretase, one of the enzymes involved in the processing of APP to amyloid beta (Hardy and Selkoe, 2002). These results suggested that production of amyloid beta and formation of senile plaques may cause AD in a direct or an indirect manner. Tau pathology may be associated with amyloid beta in the neocortex (Braak and Braak, 1997). Therefore, it is widely hypothesized that amyloid beta is an upstream causative factor in the pathogenesis of AD, which is known as the amyloid beta cascade hypothesis (Hardy and Selkoe, 2002). Alternatively, previous findings indicated that tau is essential for the pathogenesis induced by amyloid beta. For example, using human APP transgenic-mice, Roberson et al. (2007) suggested that suppressing endogenous tau effectively ameliorated amyloid beta-induced neurotoxicity; thus, tau may be a downstream effector of the amyloid beta cascade and is a potential therapeutic target for AD (Giacobini and Gold, 2013). In addition, tau is also essential for amyloid beta-induced neurotoxicity in cultured cells (Rapoport et al., 2002).

## Neurotoxic Species of Tau

Because of the association between NFT and neuronal cell loss, it is believed that formation of tau-inclusions may cause toxicity to neuronal cells via an unknown pathway. To date, the biochemical characteristics of accumulated tau (PHF-tau) have been widely studied. Some abnormal post-translational modifications have been identified, including phosphorylation, ubiquitination, glycosylation and acetylation (Mandelkow and Mandelkow, 2012). However, there is no conclusive evidence of the involvement of tau-inclusions in neurotoxicity for any of the post-translational modifications.

Another possible neurotoxic mechanism of tau was identified in pathological studies. Studies on AD in the cerebral neocortex suggested that the number of neurons lost is greater than the number of neurons affected by NFT (Gomez-Isla et al., 1997). In general, after neuronal death, NFT become ghost tangles (Braak et al., 1994). Therefore, neuronal loss without ghost tangles suggested that an NFT-independent mechanism contributed to neurotoxicity in AD brains, especially in the cerebral neocortex. Santacruz et al. (2005) suppressed tau expression in a tau-transgenic mouse model using the Tet-Off system, which effectively improved neuronal function but did not alter the number of tau-inclusions. Gilley et al. (2016) developed tau knock-in mouse lines and found that neurotoxicity was induced without tau-inclusions. Wheeler et al. (2015) also generated a human tau transgenic mouse and observed behavioral deficits in the absence of NFT-like inclusions. Moreover, human tau-A152T (a rare tau mutation) transgenic mice showed neuronal dysfunction without insoluble tau aggregates, suggesting a neurotoxic mechanism involving soluble tau (Coppola et al., 2012; Maeda et al., 2016). Analyses of *Drosophila* and *Caenorhabditis elegans (C. elegans)* also suggested that the mechanism of neurotoxicity is not always associated with the formation of NFT-like tau-inclusions, which raised the possibility that toxic species of tau do not include NFT-like tau-inclusions (Spittaels et al., 1999; Cowan and Mudher, 2013; Xie et al., 2014). In addition, Kuchibhotla et al. (2014) found that neurons bearing tau-inclusions exhibited normal activity as detected by two-photon calcium imaging.

Some hypotheses regarding specific toxic tau species have been proposed. Currently, tau oligomers are an attractive target. Lasagna-Reeves et al. (2010, 2011) prepared tau oligomers seeded by amyloid beta aggregates and used the oligomers to treat cultured cells and wild-type mice and examined neurotoxicity. Their results suggested that tau oligomers, rather than tau monomers and fibers, induced significant neurotoxicity. Furthermore, they prepared an anti-tau oligomer antibody, T22, to examine AD brains and suggested that the number of T22 positive cells was significantly higher than the normal controls. Similar results from Patterson et al. (2011) suggested that there is a higher number of cells positive for the anti-tau dimer and oligomer antibody TOC1 in AD brains than the normal controls. However, because these anti-tau antibodies are cross-reactive with tau fibers to some extent, we should be cautious in our interpretation of the role of tau oligomers in the pathogenesis of AD.

## The Neurotoxic Sequences of Tau

Because the formation of tau-inclusions takes months to a few years, neuronal dysfunction may occur before neuronal death (Miyasaka et al., 2005). Thus, to analyze tau neurotoxicity, the best choice is to examine *in vivo* models with well-characterized neural networks.

*C. elegans* is an organism that normally dwells in the soil. *C. elegans* is a widely used experimental animal because of its short lifespan, its convenient genetic manipulation and its fully sequenced genome. Moreover, approximately 42% of human disease-related genes have orthologs in the *C. elegans* genome. To date, many studies have used *C. elegans* to research neurodegenerative diseases, including tauopathy (Markaki and Tavernarakis, 2010; Li and Le, 2013).

We constructed cDNA of full-length human tau and its fragments downstream of the *unc119* promoter to overexpress tau in *C. elegans* neurons. The results suggested the aberrant behavior (uncoordinated movement, UNC) was caused by neuronal dysfunction due to tau expression. The UNC was not induced by expression of other tau-unrelated proteins such as GFP, which significantly exceeded tau expression levels. Moreover, tau expression levels influenced UNC in a dose-dependent manner and UNC was significantly worse in the FTDP-17 mutants. These results suggested that transgenic *C. elegans* is a good *in vivo* model for the study of neurotoxicity induced by tau (Xie et al., 2014; Miyasaka et al., 2016).

Biochemical analysis suggested that expressed tau in *C. elegans* is hyperphosphorylated and disassociated with microtubules, which is similar to PHF-tau. In addition, expressed tau is soluble in Triton X-100, Sarkosyl and RIPA buffer, suggesting the absence of NFT-like tau-inclusions in the *C. elegans* model. However, morphological analysis revealed aberrant tortuous processes on the nerve fibers, which suggested that neurotoxicity is independent of tau-inclusion formation (Xie et al., 2014; Miyasaka et al., 2016).

Next, we asked which sequence is responsible for tau neurotoxicity. The full-length, carboxyl terminal and amino terminal of tau were separately overexpressed in the nervous system of *C. elegans*. Then, neurotoxicity was evaluated using the percentage of *C. elegans* exhibiting UNC. Neurotoxicity was observed in tau-transgenic *C. elegans* overexpressing the full length and carboxyl terminal but not in the animals overexpressing the amino terminal. Moreover, the analysis of tau isoforms revealed that four-repeat tau induced significantly more severe neurotoxicity than three-repeat tau in transgenic *C. elegans*. These results suggested that the carboxyl terminal of tau, including MTBD, was responsible for neurotoxicity (Xie et al., 2014).

## The Carboxyl Terminal of Tau

**Figure 1:**
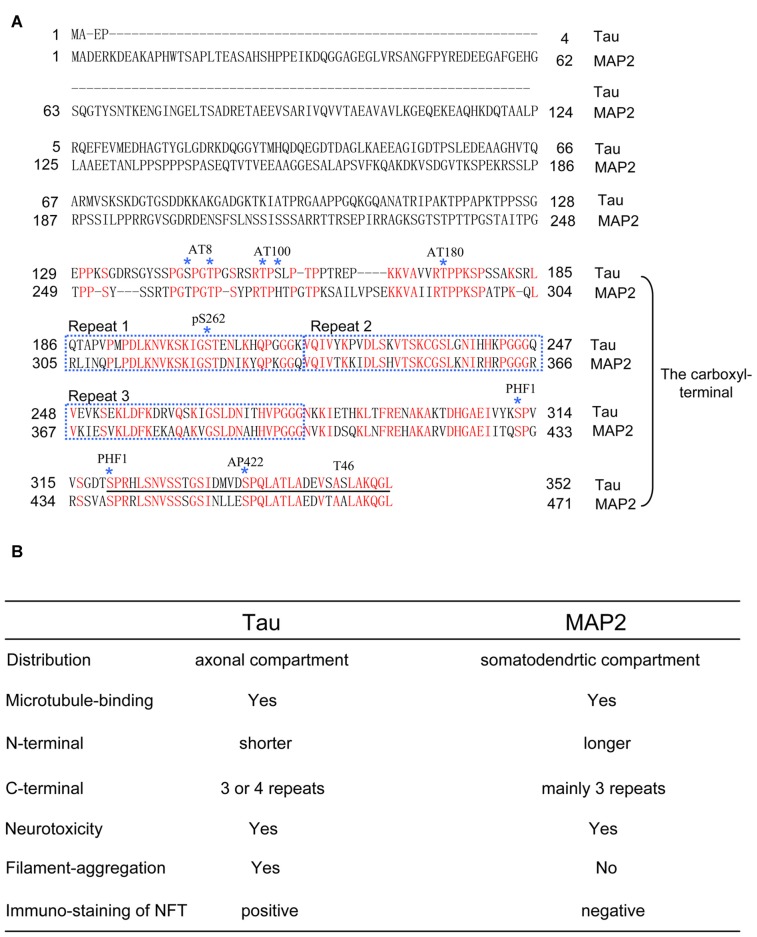
**(A)** The comparison of full-length amino acid sequences of tau (0N3R) and microtubule-associated protein 2c (MAP2c). Epitopes of anti-tau antibodies, including AT8, AT100, AT180, pS262, PHF1, AP422 and T46, are shown. Note that MAP2 also has a similar amino acid sequence with the epitopes of some antibodies that recognize the carboxyl-terminal region of tau. For consistency, in the article, the numbers of the amino acids in tau mutations and phosphorylated sites correspond to the longest tau isoform 2N4R. **(B)** Table of functional and pathological features between tau and MAP2.

The structure of tau is mainly separated into two parts, including the amino-terminal sequences and the carboxyl-terminal sequences. As a microtubule-associated protein, the carboxyl terminal of tau is notable because of the special microtubule binding sites with repeats (Figure 1). Interestingly, the carboxyl terminal of tau is also the key domain that forms the core structure of NFT under pathological conditions (Kondo et al., 1988; Wischik et al., 1988; Jakes et al., 1991). These facts suggested that there may be competition between the physiological functions and pathological aggregation of tau (Kadavath et al., 2015).

Xu et al. (2016) found that exposing the carboxyl terminal may promote tau aggregation. Additionally, a portion of the carboxyl terminal of tau is enough to form Alzheimer’s filaments (von Bergen et al., 2000). In addition to tau aggregation, MTBD is enough to induce neurotoxicity in tau pathological models (Sanders et al., 2014; Decker et al., 2015; Hochgräfe et al., 2015). Most *MAPT* mutations, such as G272V, V337M and R406W and post-translational modifications including acetylation, O-GlcNAc modification and phosphorylation, are located on the carboxyl terminal of tau (Goedert et al., 1998; Spillantini and Goedert, 2000; Mandelkow and Mandelkow, 2012; Yuzwa et al., 2012). Acetylation of K280, which is localized in MTBD, accelerates pathological tau aggregation and increases tau neurotoxicity *in vivo* (Cohen et al., 2011; Gorsky et al., 2016). Deletion of K280 strikingly enhances the aggregation propensity and cellular toxicity of tau, despite a decrease in the expression of 4R-tau (Khlistunova et al., 2006; Mocanu et al., 2008; Momeni et al., 2009). Moreover, Taniguchi-Watanabe et al. (2016) found different carboxyl-terminal band patterns in various tauopathies, indicating a novel classification for each tau-related disease. This increasing evidence provided us with insight into the close relationship between the carboxyl-terminal sequences of tau and the pathogenesis of tauopathies.

## The Potential Relationship Between MAP2 and Tauopathy

As described above, the carboxyl-terminal sequences of tau are closely involved in the pathogenesis of tauopathy. If these findings are accurate, then another question should be asked. Does microtubule-associated protein 2 (MAP2), which shares carboxyl-terminal sequences with tau, also induce neurotoxicity and associate with tauopathies? Under physiological conditions, tau is located in the axons, whereas MAP2 is located in the cell body and dendrites (Dehmelt and Halpain, 2005). In brains affected by tauopathy, the somatodendrites containing MAP2 overlap with the area where tau pathology is observed. Thus, understanding the properties of MAP2 will help us better understand the pathogenesis of tauopathy (Figure 1).

The relationship between tau and neurodegenerative diseases has been widely studied. However, few reports link MAP2 to human diseases. After tau was identified as the major component of NFT, there were different opinions on whether MAP2 was also a component of NFT. Kosik et al. (1984) prepared anti-MAP2 monoclonal antibodies and suggested that MAP2 may localize to NFT using immunochemistry methods. Whereas Rosemblatt et al. (1989) claimed that MAP2 is not located in NFT. The results of Nukina et al. (1987) suggested that the contradiction may be caused by cross-reactivity between anti-MAP2 antibodies and tau.

The amino terminal of tau projects from part of the core structure of NFT and is gradually cut off with increasing aggregation time (Miyasaka et al., 2005). In adult human brains, MAP2 is mainly expressed as a 280 kDa isoform, which has a longer amino-terminal projecting domain than tau and may be more easily removed by proteases. Therefore, although the anti-MAP2 amino-terminal antibodies are not considered to cross-react with tau, inaccurate results may be obtained due to MAP2 degradation. To solve this issue, we focused on the differences between homologous carboxyl-terminal sequences of tau and MAP2, and prepared anti-MAP2 polyclonal antibodies, which did not cross-react with tau. Using these new anti-MAP2 antibodies, we examined MAP2 localization in AD brains and found that MAP2 remained in the tangle-bearing neurons, but did not co-localize with NFT (Xie et al., 2014). Moreover, the Sarkosyl-insoluble fractions from human AD brains and normal controls were immunoblotted with tau and MAP2 antibodies. The results suggested that tau, but not MAP2, was deposited in the Sarkosyl-insoluble fractions from AD brains (Xie et al., 2014). Although tau and MAP2 share homologous carboxyl-terminal sequences, they experience different fates during NFT formation.

Why do tau and MAP2 have different fates in AD brains? To answer this question, we expressed and purified recombinant tau and MAP2 from E. coli, and investigated their aggregation properties. Tau aggregates in the presence of heparin *in vitro*, which can be detected by Thioflavin T (ThT; Goedert et al., 1996). Thus, the aggregation properties of tau and MAP2 were compared with an *in vitro* aggregation method. The results showed that the fluorescence of ThT increased by incubating tau with heparin. However, when MAP2 and heparin were incubated together, the fluorescence remained low. The aggregated fractions were further examined by atomic force microscope, and we found that tau formed fibers, whereas MAP2 formed granules. Therefore, tau and MAP2 have completely different properties of aggregation (Xie et al., 2015).

Further, we constructed, expressed and purified a series of tau and MAP2 mutants to identify their aggregation abilities using the same methods described above. The results suggested that the homologous carboxyl terminal of tau and MAP2 are the key domains that determine their aggregation properties. Exchanging three amino acids of the carboxyl-terminal sequences can reverse their fiber formation abilities. Therefore, the different aggregation properties between tau and MAP2 are only determined by three amino acids around K280 (Xie et al., 2015).

We finally used transgenic *C. elegans* to examine and compare the neurotoxicity of MAP2 and tau. The results suggested for the first time that MAP2 also induced significant neurotoxicity similar to tau. Interestingly, the key neurotoxic sequences are in the homologous carboxyl terminal of MAP2 (Xie et al., 2014). Thus, MAP2 and tau both induced neurotoxicity via their homologous carboxyl-terminal sequences *in vivo*. However, MAP2 is not deposited in NFT and does not form fibers comparable to tau (Xie et al., 2014, 2015). Therefore, fiber formation and neurotoxicity may be unrelated.

## Conclusions

**Figure 2:**
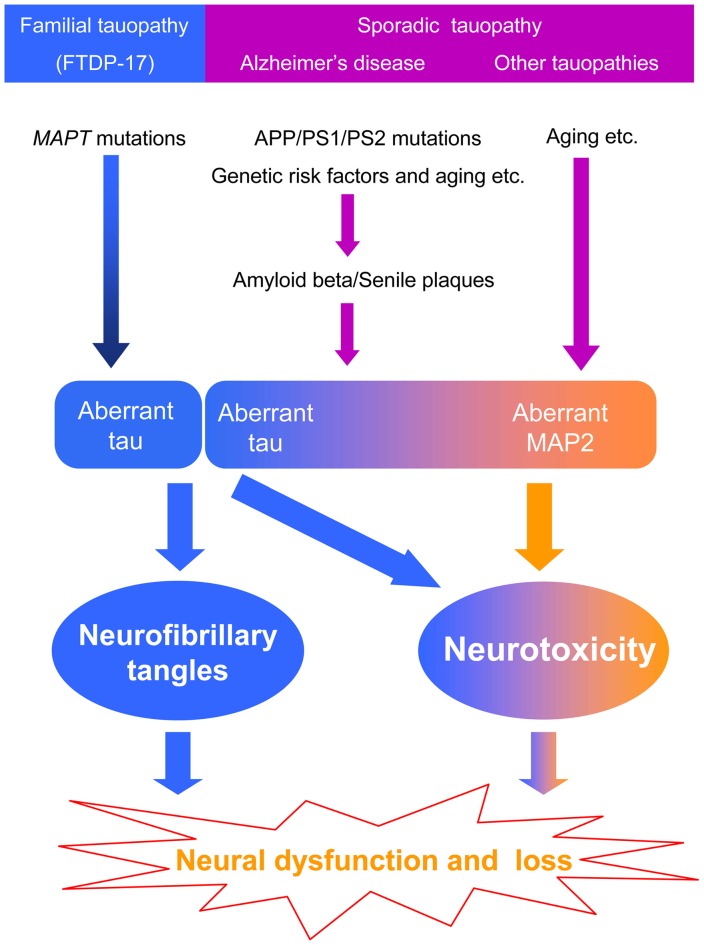
**The hypothesis of the potential involvement of MAP2 in the pathogenesis of tauopathy**.

Most of the studies on dementia focus on the proteins forming specific pathological inclusions. However, it is unknown whether the pathological inclusions are byproducts or causative factors. Currently, genetic analysis is the most powerful tool to identify causative factors in human diseases. However, mutations in tau are not found in sporadic tauopathies, including AD. We cannot exclude the possibility that the pathogenesis of familial and sporadic tauopathies is different. In familial hereditary tauopathy, aberrant tau inclusions induce neurotoxicity because of mutations in the *MAPT* gene. Whereas in sporadic tauopathy, especially in AD, mutations in APP, PS1 and PS2, aging and other risk factors produce amyloid beta that forms senile plaques and then leads to tau pathology through unknown processes. Moreover, amyloid beta toxicity mainly localizes to the somatodendritic area where MAP2 is located. MAP2 may also be involved in the pathogenesis of tauopathies, but it is difficult to find MAP2 inclusions because of its weak fiber-formation abilities. However, MAP2 can induce neurotoxicity via the homologous carboxyl-terminal sequence, which may cause neuronal dysfunction without leaving any pathological traces (Figure 2).

## Author Contributions

CX and TM wrote the article and had final approval of the submitted and published versions.

## Funding

Supported in part by Grant-in-Aid (KAKENHI) for Young Scientists (B) 20700324 to TM and 24700368 to CX and Grant-in-Aid for Scientific Research on Innovative Areas (Brain Protein Aging and Dementia Control 26117004 to TM).

## Conflict of Interest Statement

The authors declare that the research was conducted in the absence of any commercial or financial relationships that could be construed as a potential conflict of interest.
